# Organoid Models to Study Human Infectious Diseases

**DOI:** 10.1111/cpr.70004

**Published:** 2025-02-20

**Authors:** Sijing Zhu, Dan Chen, Xinzhi Yang, Liuliu Yang, Yuling Han

**Affiliations:** ^1^ Key Laboratory of Organ Regeneration and Reconstruction, State Key Laboratory of Stem Cell and Reproductive Biology Institute of Zoology, Chinese Academy of Sciences Beijing China; ^2^ Institute for Stem Cell and Regeneration, Chinese Academy of Sciences Beijing China; ^3^ University of Chinese Academy of Sciences Beijing China; ^4^ Beijing Institute for Stem Cell and Regenerative Medicine Beijing China; ^5^ State Key Laboratory of Experimental Hematology, National Clinical Research Center for Blood Disease, Haihe Laboratory of Cell Ecosystem Institute of Hematology & Blood Diseases Hospital, Chinese Academy of Medical Sciences & Peking Union Medical College Tianjin China; ^6^ Tianjin Institute of Health Science Tianjin China

**Keywords:** drug screening, infectious disease, organoids, virus

## Abstract

Infectious diseases have become significant events that threaten global public health and economic development. Since the 20th century, multiple outbreaks of infectious diseases have gradually deepened humanity's understanding of viral infections, prevention and treatment. Organoids possess a high degree of similarity to human physiological states and have strong self‐organising capabilities. Research on infectious diseases based on organoids offers significant advantages in terms of availability, editability and diversity. In this perspective, we briefly introduce the development of organoids, focusing on historically significant infectious diseases that have caused fatal harm to human health, such as HIV, ZIKV, SARS‐CoV‐2 and MPXV. We further summarise relevant research on the pathogenic mechanisms of these viruses based on organoid models, host reactivity, and therapeutic strategies. Finally, we list the latest research techniques combined with organoid models, discuss the challenges faced in the development of organoids and look forward to the future prospects of organoids in vaccine and drug development.

## Graphical abstract

Our manuscript reviews the role of organoids as models for studying human infectious diseases, highlighting their irreplaceable contributions to drug testing and vaccine development for significant infectious diseases including HIV, ZIKV, SARS‐CoV‐2 and MPXV. The integration of organoids with new technologies will create broader opportunities for the advancement of regenerative medicine.

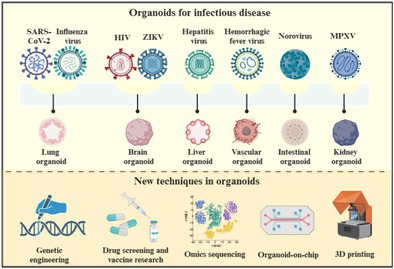

## 1 Origin and History of Organoid Models

Organoids refer to tissue‐like structures that are self‐organised through the in vitro three‐dimensional (3D) culture of adult stem cells or pluripotent stem cells (PSCs), possessing a certain spatial architecture. Organoid‐based disease models offer significant advantages in terms of editability and diversity, as they can not only simulate the changes occurring in different tissues and cells during physiological and pathological processes, aiding research on infectious diseases, genetic disorders, tumours and organ development but also integrate gene editing and high‐throughput sequencing technologies, providing robust technical support for disease modelling and drug development.

**FIGURE 1:**
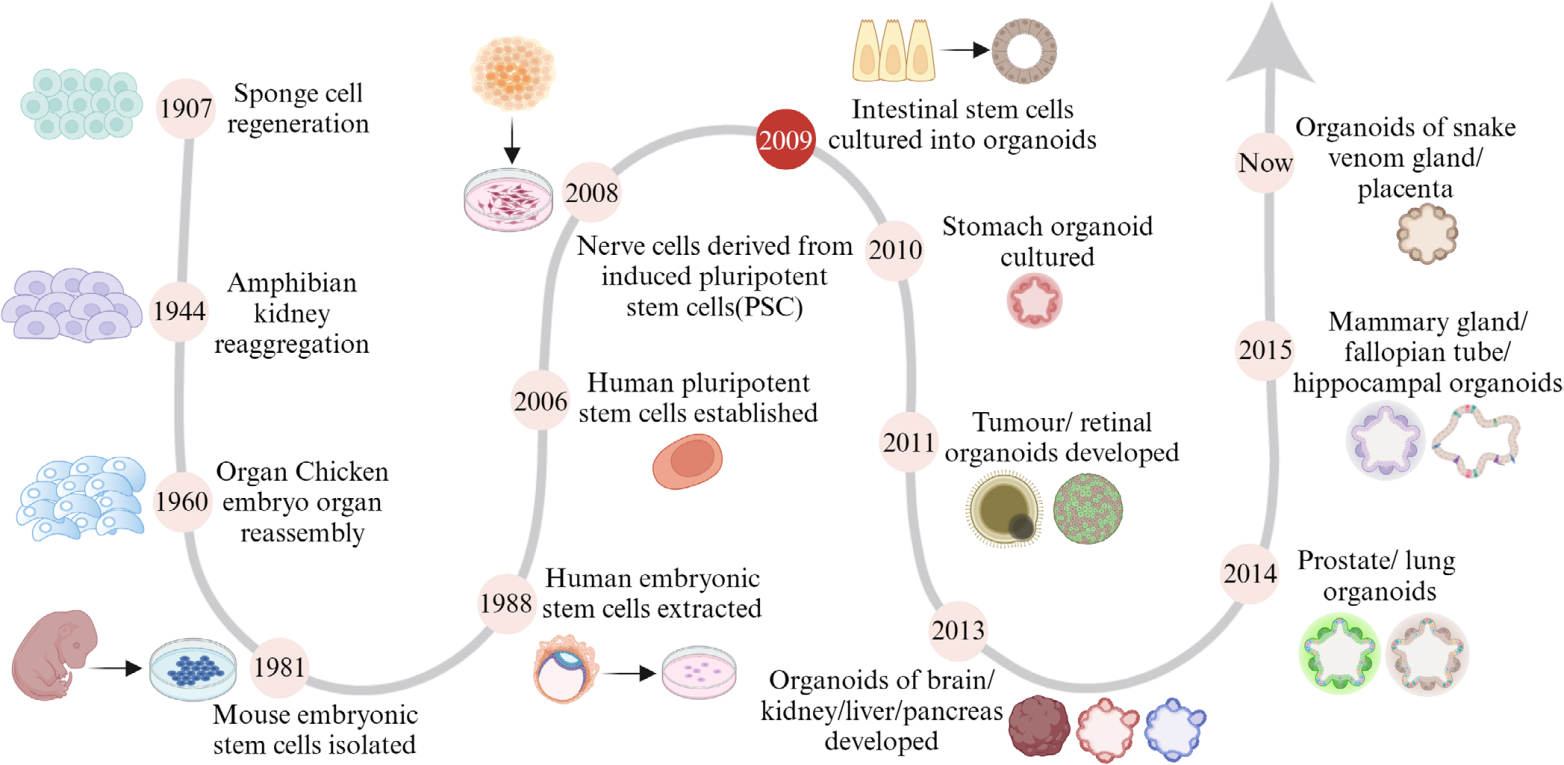
The research process of establishing different kinds of organoid models. This diagram shows important events in the development of organoids from their birth to the present.

The development of organoids can be divided into three stages. The early exploration stage began in 1907 when Henry Van discovered that sponge cells could reaggregate into a complete organism through self‐organisation [1]. In 1944, Holtfreter's experiment on the isolation and aggregation of amphibian pronephros tissue and in 1960 Weiss's experiment on the isolation and aggregation of multiple organs of chicken embryo found that similar self‐organisation phenomena also existed in vertebrates [2]. In early studies of 3D cell culture in 1965, organoids were thought to be abnormally grown cells [3]. PSCs were first isolated from mouse embryos by Evans in 1981 [4], while human embryonic stem cells (ESCs) were first isolated from human blastocysts by scientist James Thomson in 1998 [5]. Subsequently, in 2006, the protocol for human induced pluripotent stem cells (iPSCs) was successfully constructed by defined factors, greatly expanding the cellular sources for organoid culture [6]. In 2008, Eiraku et al. showed the process by which iPSCs in the human brain self‐organised into nerve cells that form polarised cortical tissue [7]. The vigorous development of organoid technology began in 2009, when Hans Clevers and his colleagues successfully cultured a single Lgr5^+^ intestinal stem cell into an intestinal crypt‐villus structure organoid containing all intestinal cell types by simulating the intestinal microenvironment in vitro [8]. It was found that overactivation of WNT signalling pathway and inhibition of BMP pathway were important for intestinal stem cells to maintain self‐renewal. This research has established organoid as a promising model to replace traditional cell lines and heterogeneous animal models, and the construction of different kinds of organs has mushroomed. Following this, the construction of various organoids, such as those from the stomach [9], retina [10], brain [11], kidney [12], liver [13], pancreas [14], prostate [15, 16], lung [17], breast [18], fallopian tube [19] and placenta [20] have emerged rapidly (Figure 1). As early as 2012, organoid models had been applied to the study of infectious diseases [21], and in 2017, they were named one of the top 10 technological breakthroughs, marking the beginning of a new era in the development of organoids and regenerative medicine. Research based on brain organoids exposed to Zika virus suggests the neurotropism of ZIKV [22]. An HIV infection model established using tonsil organoids reveals the mechanisms by which chronic viral infection causes T‐cell dysfunction [23]. A monkeypox virus (MPXV) infection model established using skin organoids identified the potential antiviral drug tecovirimat for MPXV [24]. Organoid models have been extensively utilised in numerous areas, including drug development, gene editing, organ repair, tumour immunotherapy and personalised medicine.

## 2 Organoids to Study Infectious Diseases

**FIGURE 2:**
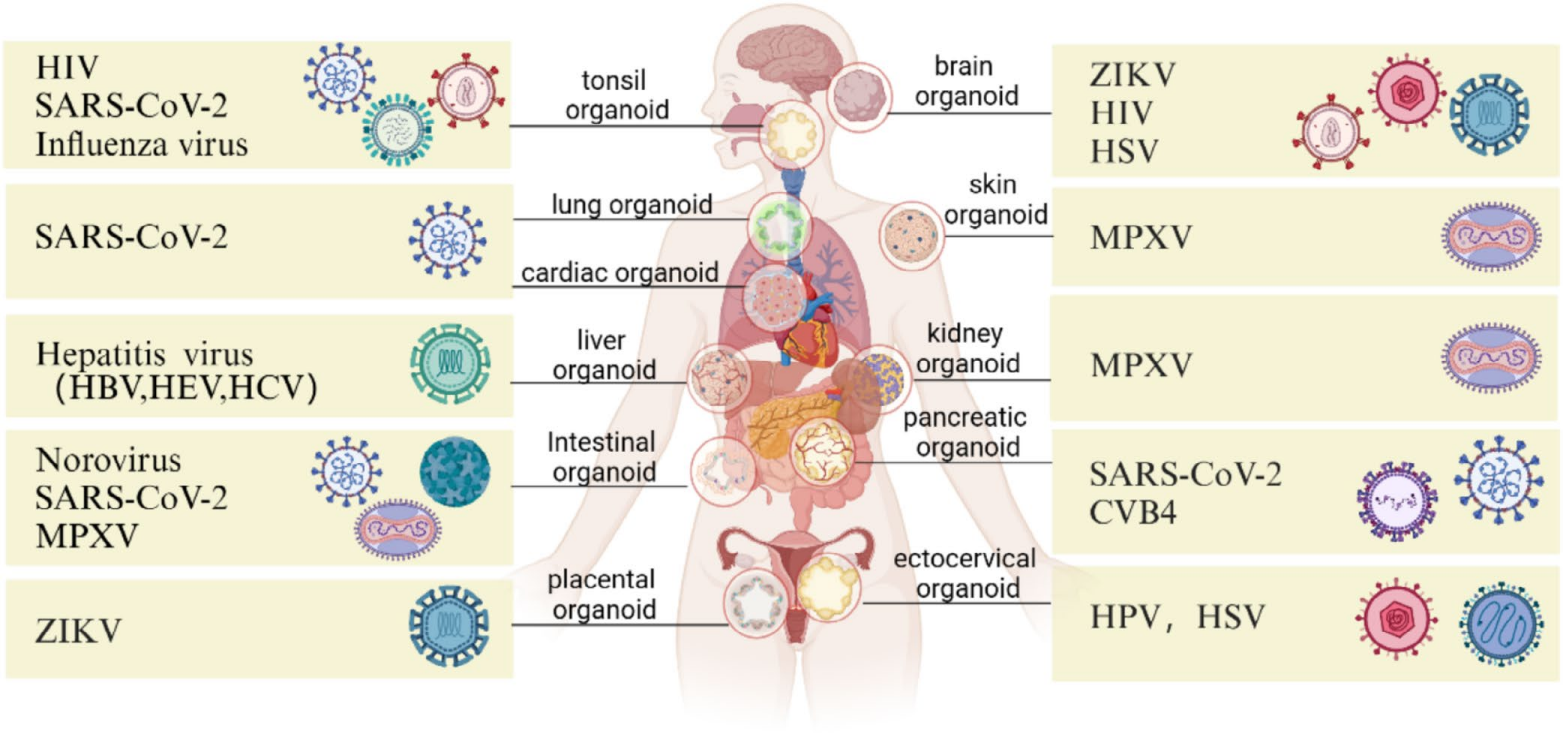
Application of different organoids in virus infectious disease. In human history, a variety of epidemic viruses have caused varying degrees of damage to a variety of organs, including brain, lung, heart, liver, kidney and other important organs.

Infectious diseases, especially viral infectious diseases such as HIV, ZIKV, SARS‐CoV‐2, MPXV, and so forth, pose a major threat to global public health. However, there are some limitations of traditional two‐dimensional (2D) cell culture and animal models in studying the interactions between pathogens and host cells, especially the mechanisms of infection and their pathological processes. 2D cell culture cannot completely reproduce the microenvironment of human organs and the complex interactions between cells. Although animal models provide a research platform closer to the human body, there are many problems in animal welfare, cost, and species differences. However, organoids can be constructed from adult stem cells or PSCs derived from patients, eliminating species differences. The 3D culture method more authentically simulates the microenvironment and tissue structure of human organs. Organoid disease models not only help us gain a deeper understanding of the pathogenic mechanisms of viruses but also provide an important platform for antiviral drug screening and vaccine development, significantly enhancing the efficiency of drug research and development and improving clinical treatment outcomes (Table 1, Figure 2).

**TABLE 1:** Summary of the application of organoids to study infectious disease.

Organoid model	Cellular sources	Major cell types	Susceptibility
Brain	hPSC/ESC	Neural progenitor cells, neurons, astrocytes, oligodendrocytes	HIV [25], ZIKV [26, 27], SARS‐CoV‐2 [28, 29], HSV [30]
Choroid plexus	hPSC/ESC	Epithelium, neurons, fibroblast, endothelial, immune	
Nasal cavity	Adult	Basal cell, ciliated cell, goblet cell, secretory cell, mucosal glandular cell	SARS‐CoV‐2 [31, 32]
Tonsil	hPSC/ESC	B cell, T cell, plasmablasts, natural killer cells, Treg cell, dendritic cell, epithelium	HIV [23], SARS‐CoV‐2 [31], Influenza virus [33]
Salivary gland	hPSC/ESC, adult	Basal cell, ductal cell, acinar cell, myoepithelial cell, fibroblasts	SARS‐CoV‐2 [34]
Bronchial tubes	hPSC/ESC, adult	Goblet cell, ciliated cell, basal cell, club cell, epithelium	SARS‐CoV‐2 [35, 36]
Alveolar	hPSC/ESC, adult	AT2/AT1 epithelium cell, basal cell, secretory cell	
Cardiac	hPSC/ESC	Cardiomyocytes, valve cell, proepicardial‐derived cell, epicardial cell, epicardial cell, conductance cell	SARS‐CoV‐2 [37]
Liver	hPSC/ESC, adult	Hepatocytes, cholangiocytes, hepatic stellate cell, hepatic sinusoidal endothelial cell	HBV [38, 39], HCV [40], HEV [41]
Kidney	hPSC/ESC	Glomerular progenitor cell, ureteric epithelial progenitor cell, tubular epithelial cell, podocytes, parietal epithelial cell, macula densa cell	MPXV [42]
Gastric	hPSC/ESC, adult	LGR5^+^ stem cell, gastric endocrine cell, epithelium	SARS‐CoV‐2 [43]
Skin	hPSC/ESC, adult	Keratinocytes, melanocytes, fibroblasts, sweat gland cell, sebaceous gland cell, adipocytes	MPXV [24]
Capillary	hPSC/ESC	Endothelial cell, smooth muscle cell, pericytes, mesenchymal	EBOV [44]
Intestine	hPSC/ESC, adult	Paneth cell, goblet cell, enteroendocrine cell, tuft cell, BEST4^+^ enterocytes	MPXV [45], SARS‐CoV‐2 [36], HuNoV [46, 47]
Colonic	hPSC/ESC, adult	Goblet cell, enterocytes, endocrine cell, paneth cell	
Pancreatic	hPSC/ESC, adult	α/β/δ/PP/γ cell, acinar cell, ductal cell, endocrine cell, LGR5^+^ stem cell, duct cilium cell	SARS‐CoV‐2, CVB4 [48]
Testicular	Adult	Sertoli cell, leydig cell, peritubular myoid cell	ZIKV [49]
Placental	hPSC/ESC	Trophoblast cell, mesenchymal cell, endothelial cell	ZIKV [50]
Ectocervical	Adult	Basal cell, intermediate cell, superficial cell, columnar epithelial cell	HPV [51]

### 2.1 Organoid Models for HIV Research

The human immunodeficiency virus (HIV), a retrovirus, was first identified in the United States in 1983. HIV attacks the human immune system by destroying CD4^+^ T lymphocytes, leading to immune system collapse, which can progress to acquired immune deficiency syndrome (AIDS) and result in various severe opportunistic infections and tumours [52]. The specific mechanism by which HIV infects CD4^+^ T lymphocytes involves recognising its surface co‐receptors, CCR5 (C‐C chemokine receptor type 5) or CXCR4 (C‐X‐C chemokine receptor type 4), and binding to these sites to enter the host cells [53]. Currently, there are 38 million people worldwide infected with HIV, resulting in millions of deaths. Although antiretroviral therapy (ART) has significantly reduced HIV‐related mortality, developing an effective vaccine and finding a cure for the disease remain challenging, making HIV a major global health challenge [54].

*The application of organoids in HIV research*: HIV is both lymphotropic and neurotropic. The HIV infection model in human tonsil organoids revealed that HIV‐infected T cells increase polyamine synthesis intermediates and upregulate ornithine decarboxylase‐1 (ODC1), causing an imbalance between TregDys and Th17 cells—a finding consistent with patient observations. This reveals the mechanism by which chronic viral infection drives T‐cell effector programmes and the dysfunction of regulatory T cells [23]. In addition to primarily infecting CD4^+^ T lymphocytes, HIV can also infect monocytes, B lymphocytes, natural killer cells and microglia. Brain and choroid plexus organoid studies revealed that HIV‐infected microglial cells upregulate chemokines CCL2 and CXCL10, along with type I interferon (IFN)‐stimulated genes and members of the S100 inflammatory gene family. These inflammatory factors interact with uninfected neurons through multicellular communication, disrupting the function and survival of normal neurons. This suggests that HIV infection of microglial cells in the brain is a significant contributor to HIV‐associated neurocognitive disorder (HAND) [25]. Coincidentally, another study found that neurogranin (*NRGN*) may also play an important role in the development of HAND, as it is significantly downregulated at both the mRNA and protein levels in the context of HIV infection. The inflammatory factors released by HIVinfected microglial cells can lead to the proliferation of astrocytes. These changes further exacerbate neuroinflammation, resulting in neuronal damage [55].

*AIDS treatment regimen*: AIDS treatment strategies encompass both curative and functional approaches, and achieving a complete cure will require more time. Currently, the commonly used medications for combating HIV globally include Tenofovir alafenamide fumarate, HIV protease inhibitors and rilpivirine [56]. One of the more effective treatment methods is ART, also known as ‘cocktail therapy’, but it necessitates long‐term and frequent medication adherence [57]. Researchers are exploring new avenues, such as a study by Satya Dandekar and colleagues, who utilised a simian model of AIDS in rhesus monkeys. They found that the mesenchymal stem cells derived from the bone marrow could reduce the viral load responsible for AIDS, enhance antiviral immunity, and repair and restore gut lymphoid follicles damaged by simian immunodeficiency virus (SIV), providing a supplementary treatment approach for AIDS [58]. Dolutegravir (DTG), an integrase strand transfer inhibitor, is widely used in ART. However, its use during pregnancy may increase the risk of neural tube defects (NTDs) in the foetus. Brain organoid models have also facilitated the assessment of drug efficacy for AIDS treatment. Studies have showed that DTG exposure upregulated FOLR1 and altered neurogenesis‐related genes (DVL3, GATA2, KIF22), alongside changes in organoid biomechanical properties [59]. Due to technical limitations, organoids can only simulate a single brain region rather than complex brain tissue in most cases. Future research needs to optimise culturerapidly conditions to construct more mature and complex brain tissue structures, thus providing a robust platform for screening and evaluating HIV drugs that can cross the blood–brain barrier and exert effects within the central nervous system (CNS).

### 2.2 Organoid Models for ZIKV Research

The Zika virus (ZIKV) is a single‐stranded positive‐sense RNA virus that rampantly spread in South American in 2015, causing infections in over 1.5 million people and becoming an international public health emergency. The *Aedes aegypti* mosquito is the main vector for the transmission of ZIKV. It spreads widely after cross‐mosquito bites. ZIKV effectively infects neural stem cells during the first trimester of pregnancy, leading to apoptosis of these cells and damage to brain tissue. This ultimately results in congenital neurological defects and severe microcephaly (commonly known as ‘small head syndrome’) in the foetus. In adults, ZIKV infection can also result in Guillain‐Barré syndrome [60]. The viral glycoprotein E is crucial for mediating the entry of ZIKV into cells, followed by infection of the cells through the synergistic action of clathrin and acidic endosomes [61]. Research has found that when ZIKV non‐structural proteins NS1, NS4B, and NS2B3 inhibit induction of IFN and downstream IFN‐stimulated genes using various strategies, it triggers ZIKV evasion of the host's antiviral response [62].

*The application of organoids in ZIKV research*: Organoid models have been applied to study ZIKV infection. Researchers exposed forebrain organoids to an environment containing ZIKV using a rotatable, multi‐well device called SpinΩ. They found that neural progenitor cells (NPCs) within the organoids could be effectively infected, resulting in an increased cell death rate, a significant reduction in the thickness of the ventricular zone (VZ) and the neural layer, which exacerbated the damage to the cortical structure of the brain [22]. Moreover, ZIKV infection can induce neurological complications such as epilepsy. The TRPC channels play a crucial role in regulating neuronal excitability. Chen and colleagues discovered that infection by the ZIKV leads to an upregulation of TRPC4 in host cells. This upregulation is facilitated by the interaction between the ZIKV‐NS3 protein and CaMKII, which in turn augments the calcium influx that is mediated by TRPC4. The study also indicated that suppressing CaMKII results in a decrease in the levels of both pCREB and TRPC4 proteins. Furthermore, it was observed that the inhibition of either TRPC4 or CaMKII can enhance the survival rate of cells infected with ZIKV and can also lead to a reduction in the synthesis of viral proteins. This process may interfere with the replication stage of the viral lifecycle and have a positive therapeutic effect on epilepsy induced by ZIKV infection [63]. In addition to impacting brain development, ZIKV can also breach the blood‐placental barrier, damaging human trophoblast stem cells (hTSCs) and infecting the foetus through vertical transmission. The study by Wu et al. indicates that ZIKV infection triggers a significant activation of the antiviral genes IFITs and IFITMs, resulting in the disruption of the structure of trophoblast organoids derived from human trophoblast stem cells, thereby inhibiting the formation of syncytia and affecting normal placental development [50]. Similarly, ZIKV can penetrate the blood‐testis barrier, infecting testicular cells and facilitating sexual transmission. Yang et al. used primary testicular cells from adult BALB/c mice to construct a 3D testicular organoid model. They found that the testicular organoid had a blood‐testis barrier‐like structure and testosterone synthesis capabilities similar to the testes. ZIKV effectively established infection in the mouse testicular organoid and impaired the blood‐testis barrier and its physiological functions, which is highly consistent with the pathological manifestations of testicular infection in mammals [49].

*Zika Virus Disease treatment regimen*: Currently, the strategies for the discovery of ZIKV inhibitors mainly include screening diverse libraries of small molecules or natural compounds, repurposing known active compounds or clinical drugs through drug repositioning, and designing antibody‐based candidate drugs. Researchers conducted high‐throughput chemical screening using hNPCs and found that Hippeastrine hydrobromide and Amodiaquine dihydrochloride dihydrate effectively inhibit ZIKV infection in hNPCs. Hippeastrine hydrobromide also rescues the growth and differentiation defects induced by ZIKV in hNPCs and human foetal‐like forebrain organoids [64].

### 2.3 Organoid Models for SARS‐CoV‐2 Research

Coronavirus Disease 2019 (COVID‐19) is an infectious disease caused by Severe Acute Respiratory Syndrome Coronavirus 2 (SARS‐CoV‐2) that has infected more than 629 million people worldwide. Nearly 6.6 million people died, making it the most widespread and longest pandemic in recorded human history. The classic route of SARS‐CoV‐2 infection is through Angiotensin‐Converting Enzyme 2 (ACE2) on the cell surface. After binding to the receptor, SARS‐CoV‐2 mediated the cleavage of spike proteins via Transmembrane Protease Serine 2 (TMPRSS2) [65] and Cathepsin L (CTSL) [66] to promote viral membrane fusion with cells. Neuropilin‐1 (NRP1) has also been shown to promote SARS‐CoV‐2 infection [67]. In addition, studies based on pulmonary organoids have found that SARS‐CoV‐2 can also enter lung cells through macropinocytosis, a non‐classical endocytosis pathway and lung surface protein B (SP‐B) plays an important regulatory role in host immune response and cell apoptosis [35]. Because of the wide distribution of ACE2 receptor in mammalian cells, SARS‐CoV‐2 can infect the human body through the respiratory tract and rapidly spread to other organ systems. As a result, COVID‐19 patients not only exhibit acute respiratory syndrome but also cause excessive inflammatory responses and extensive multi‐organ damage [68]. Different international teams have constructed human organoids such as the intestine, whole brain, choroid plexus, nasal cavity, respiratory tract, kidney and heart based on adult stem cells or PSCs to study the tendency and in situ pathogenicity of SARS‐CoV‐2.

*The application of respiratory organoids in SARS‐CoV‐2 research*: SARS‐CoV‐2 primarily targets epithelial cells in the respiratory system, leading to symptoms such as excessive mucus secretion, coughing, and respiratory distress. The construction of respiratory organoids is mainly derived from epithelial stem cells isolated from mature lung or nasal tissue and stem cells. Han et al. used cultured organoids to simulate the inflammatory changes in lung infections caused by COVID‐19, confirming viral infection through RNA sequencing. They then performed high‐throughput screening of therapeutic agents against SARS‐CoV‐2, identifying several inhibitors, including imatinib. This indicates that hPSC‐derived lung organoid models infected with SARS‐CoV‐2 could serve as high‐throughput screening platforms, providing a potential avenue for the rapid and efficient identification of candidate therapeutics for COVID‐19 [36]. Wu et al. found that in nasal epithelial organoids infected with SARS‐CoV‐2, ciliary cells use ciliary structures to attach to the cell surface and promote infection by specific cell connection targets or by disrupting tight connections between adjacent epithelial cells, which then spread to other cells under the oscillations of the microvilli. Researchers used short hairpin RNA (shRNA) to knock down the cilia formation gene CEP83, and found that the downregulation of CEP83 significantly inhibited SARS‐CoV‐2 infection, confirming the important role of cilia in viral entry into cells. Treatment of nasal organoids with Ciliobrevin D, a ciliate kinetic protein inhibitor, showed that ciliate protein or receptor mediated the reverse transport of SARS‐CoV‐2 from ciliate tip to cell was reduced, and the infected cells of the organoids were significantly reduced, confirming the important role of ciliate protein in virus‐infected cells. At the same time, SARS‐CoV‐2 infection activates the expression of several kinases in host cells, such as PAK1 and PAK4, which phosphorylate downstream target proteins to regulate the dynamics of microvilli, promoting viral release and intercellular infection [31]. This study innovatively proposes a short‐term nasal spray medication strategy to delay viral entry and transmission, providing the immune system with more time to react and preventing the occurrence of severe infections, in addition to traditional therapeutic strategies aimed at blocking virus‐host cell binding and inhibiting the replication of new viruses in host cells.

*The application of other organoids in SARS‐CoV‐2 research*: Although SARS‐CoV‐2 primarily infects the respiratory system, COVID‐19 patients often present with symptoms related to gastrointestinal, cardiovascular and nervous systems, exhibiting life‐threatening signs such as diarrhoea, vomiting, headaches, loss of taste, myocarditis and even seizures. Researchers generated gastric organoids from biopsies of foetal, paediatric and adult gastric tissues, and after inducing the organoids to reverse polarity, exposed them to a SARS‐CoV‐2 environment. They found that viral replication was significantly lower in undifferentiated organoids derived from early foetal and adult tissues, while gastric organoids from children and late‐stage foetuses were more susceptible to SARS‐CoV‐2 infection. Additionally, differentiated adult gastric organoids were more easily infected, suggesting that SARS‐CoV‐2 may be transmitted via the faecal‐oral route [43]. Tanaka et al. constructed human salivary gland organoid models using hiPSCs that closely resemble physiological functions, discovering that SARS‐CoV‐2 can infect and replicate within these salivary gland organoids. They proposed that salivary glands may serve as a ‘reservoir’ to facilitate the spread of the virus [34]. The study established a disease model of SARS‐CoV‐2 infection in cardiac organoids (hCOs) derived from hiPSCs. Transcriptomic data analysis revealed dysregulation of genes related to endothelial damage, myocardial contraction and pro‐inflammatory factors in the infected hCOs, leading to functional impairment of both myocardial and endothelial cells [37]. The Ernst J Wolvetang team utilised brain organoid models generated from Down syndrome and heterokaryotype iPSCs to reveal the critical role of choroid plexus epithelial cells in SARS‐CoV‐2 infection. The choroid plexus defects in patients with Down syndrome promote the neurotropism of SARS‐CoV‐2. Inhibition of TMPRSS2 and furin activity effectively reduces viral replication in Down syndrome organoids [28]. Additionally, recent studies have explored SARS‐CoV‐2 infection in various cell types and organoids derived from hPSCs. Through systematic analysis of transcriptional changes, a mechanism of SARS‐CoV‐2 infection regulated by the nuclear transcription factor CIART was identified. CIART deficiency downregulated the RXR pathway, leading to decreased fatty acid synthesis and blocking SARS‐CoV‐2 infection in a cell‐independent manner, providing new directions and strategies for the development of antiviral drugs [69].

*COVID‐19 treatment regimen*: A research team conducted high‐throughput antiviral drug screening based on organoid models, as well as the evolution and pathogenesis of SARS‐CoV‐2 variant strains. Li et al. found that the Spike protein of the BA.5 variant can enhance the invasion efficiency of the SARS‐CoV‐2 and promote viral spread between cells by forming syncytia, exhibiting significantly increased replication capacity and infectivity in human nasal and airway organoids [70]. The Kwok‐Yung Yuen team compared the adaptability and immune evasion characteristics of SARS‐CoV‐2 variants EG.5.1 and XBB.1.9.1 using human nasal organoids and serum from elderly individuals, discovering that EG.5.1 possesses stronger immune evasion capabilities than XBB.1.9.1, though there were no significant differences in replication capacity [32]. Recent research has constructed computational models by integrating organoid single‐Cell RNA Sequencing (scRNA‐seq) data, expression quantitative trait loci, GWAS summary statistics, and gene‐drug interaction data. The study predicts significant differences in the severity of SARS‐CoV‐2 infection among populations with different genetic backgrounds [71]. At the moment, remdesivir alone and its combination with ciclesonide, nelfinavir, and camostat have proven to possess antiviral properties against SARS‐CoV‐2 within adult alveolar spheres [72]. Some intramuscular antiviral vaccines, including inactivated vaccines, nucleic acid vaccines, carrier vaccines, subunit vaccines, virus‐like particle vaccines, and live attenuated vaccines have been successfully developed [73]. Chen et al.'s attenuated virus‐based intranasal COVID‐19 vaccine provides rapid, durable and widespread protection against SARS‐CoV‐2 infection [74]. However, researchers have demonstrated using human pluripotent stem cell‐derived cardiomyocytes (hCMs) and human engineered heart tissue (hEHT) models that under clinically relevant concentrations, four antiviral drugs—aprimod, remdesivir, ritonavir and lopinavir—exhibit certain cardiotoxic effects. Cefepime hydrochloride, astaxanthin, and fumaric acid quetiapine can mitigate the cardiotoxicity induced by remdesivir, suggesting that we need to pay attention to drug side effects and implement appropriate protective measures during clinical treatment [75].

### 2.4 Organoid Models for MPXV Research

Monkeypox virus (MPXV) is a double‐stranded DNA virus belonging to the genus Orthopoxvirus, highly homologous to the vaccinia virus of the same genus. MPXV exists in two forms: the extracellular enveloped virus (EEV) and the intracellular mature virus (IMV), both of which are infectious forms. MPXV releases its viral core into host cells via endocytosis or membrane fusion, initially proliferating in connective tissue cells. Genes associated with the Golgi‐associated retrograde protein (GARP) complex contribute to MPXV infection, but its cellular receptors have yet to be identified. Direct or indirect animal contact is the main transmission route of MPXV, causing symptoms like fever, inflammation and skin/mucosal lesions [76]. Since the discovery of MPXV in 1958, it has long been regarded as an endemic zoonotic disease transmitted through contact with host rodents. However, research has revealed that the West African B.1 lineage IIb MPXV from 2022 has been spreading through human‐to‐human transmission since 2016, displaying phylogenetic differences from earlier endemic MPXV strains (lineages I or IIa). Notably, APOBEC3 editing is a characteristic feature of human MPXV infections [77].

*The application of organoids in MPVX research*: Organoid models have significant application value in the study of MPXV infection and treatment. Watanabe et al. successfully constructed colon organoids and exposed them to Zr‐599 MPXV, Liberian MPXV and 2022 MPXV environments for infection studies. Transcriptome sequencing revealed that compared to skin keratinocytes infected with MPXV, colon organoids infected with Zr‐599 MPXV exhibited significant expression changes in 85 genes, which were primarily enriched in pathways related to ‘zinc ion homeostasis’. Fluorescence results showed a consistent decrease in the protein level of MT1G, indicating that Zr‐599 MPXV induces gastrointestinal disease by disrupting zinc homeostasis, resulting in inflammatory responses and cell damage [45]. The study found that MPXV can effectively infect kidney organoids, leading to loss of glomerular structural integrity and disintegration of proximal tubule structures [42]. Moreover, Li et al. successfully modelled tissue structures such as the epidermis, dermis, and hair follicles from human skin organoids generated from iPSCs, which closely resemble the physiological structure of human skin. Infection of skin organoids with MPXV resulted in the upregulation of inflammatory factors in keratinocytes and recruitment of immune cells, triggering immune responses that ultimately led to cell necrosis and shedding, fully recapitulating the process of MPXV infection in human skin [24].

*Monkeypox virus disease treatment regimen*: Drug screening based on skin, kidney and brain organoids has revealed that the antiviral drug Tecovirimat shows significant effectiveness in suppressing MPXV replication and reducing pathological manifestations. Currently, Cidofovir, Brincidofovir and Tecovirimat, which are clinically used, are antiviral drugs developed against the MPXV lifecycle. Organoid models and in vitro studies have shown that Cidofovir and Brincidofovir exert anti‐MPXV effects by inhibiting viral DNA polymerase, while Tecovirimat is clinically used as an FDA‐approved treatment for MPXV and has demonstrated suitable efficacy. The nucleoside analogue inhibitor *N*‐Methanocarbathymidine (NIOCH‐14) exhibits antiviral activity and shows similar effects to Tecovirimat as a candidate drug [76]. Research has shown that natural immunity induced by three routes of MPXV infection—intravenous injection, intradermal injection and rectal injection—can provide protective effects against subsequent attacks by the MPXV [78]. Therefore, several vaccines have been developed, including the attenuated live vaccine ACAM2000 [79], the non‐replicating live virus vaccine JYNNEOS [80], the A35R extracellular domain‐M1R fusion proteins (VGPox 1 and VGPox 2), and a mixture of full‐length mRNA of A35R and M1R (VGPox 3) [81].

### 2.5 Organoid Models for Hepatitis Virus Research

*Hepatitis virus*: Hepatitis is a liver disease caused by various pathogenic factors, including viruses, bacteria, parasites, drugs, chemicals, alcohol and autoimmune disorders, with viral infection being the most common cause. The main types of hepatitis viruses include Hepatitis A (HAV), Hepatitis B (HBV), Hepatitis C (HCV), Hepatitis D (HDV) and Hepatitis E (HEV). Viral hepatitis is the second largest infectious disease affecting global health, with approximately 350 million people infected worldwide, and over a million deaths each year due to related diseases, with HBV and HCV having higher mortality rates [82]. HAV and HEV are transmitted via the gastrointestinal tract through contaminated hands, food or water, while HBV, HCV and HDV are mainly transmitted through mother‐to‐infant, sexual contact and blood transfusions. Human tumour cell lines fail to reflect liver physiology accurately, and humanised mouse models are too complex for high‐throughput drug screening. Organoid models offer a novel platform for studying viral hepatitis. Researchers have successfully established infection of liver organoids using recombinant HBV, simulating typical physiological processes of HBV infection, such as the expression of HBV e Antigen (HBeAg), generation of covalently closed circular DNA (cccDNA) and the production of infectious HBV particles. After treating infected liver organoids with Tenofovir, it was found that Tenofovir could inhibit the reverse transcription process of HBV pregenomic RNA to DNA [38]. Treatment with Tapasin shRNA revealed that Tapasin reduced autophagy via the mTOR pathway, inhibiting CD8^+^ T‐cell differentiation and ultimately hindering viral clearance in liver organoids [39]. Long‐term pharmacological treatment of viral hepatitis can cause liver damage; however, there has been a lack of good research models. Zhang et al. utilised human liver organoids in a 384‐well plate system to enable rapid screening for the risk of drug‐induced liver injury and conducted toxicological assessments on the mechanisms of hepatotoxicity, providing a comprehensive platform for understanding drug‐induced liver damage [83].

Research on HCV has revealed that the mechanism of HCV entry into cells is through binding to cell surface receptors (SR‐B1, CD81 and EGFR), migrating from the basement membrane to tight junctions, and then entering cells via endocytosis mediated by clathrin in an EGFR‐dependent manner. Polarised Huh‐7.5 hepatocellular carcinoma organoids exhibit high susceptibility to HCV, but the use of the EGFR inhibitor Erlotinib and short hairpin RNA (shRNA) can inhibit HCV infection [40]. Research by Li et al. confirmed that human liver‐derived organoids support the entire lifecycle of HEV infection. By culturing a monolayer of organoids in a polarised manner, HEV particles were observed to be released via apical secretion. Drug screening using liver organoids has identified Brequinar and Homoharringtonine as effective HEV inhibitors, which also effectively combat the ribavirin‐resistant variant harbouring the G1634R mutation [41]. By generating organoids from iPSCs with different genetic backgrounds, it is possible to compare HBV susceptibility under different risk factors within the same population or to compare HBV susceptibility among different populations under the same risk factors. For instance, different ethnic groups exhibit varying susceptibilities to hepatitis viruses; Chinese individuals are highly susceptible to HBV, while those from Europe and America are more susceptible to HCV [84].

### 2.6 Organoid Models for EBOV Research

*Haemorrhagic fever virus*: The Ebola virus (EBOV), which emerged in 1976, and the Marburg virus (MARV), which originated in 1967, both belong to the haemorrhagic fever viruses and are two of the deadliest infectious disease pathogens. Early infection is characterised by sudden high fever, headache and diarrhoea, while late‐stage symptoms include bleeding from the mouth, nose, and other areas. The incubation period for these viruses ranges from 2 to 21 days, with a mortality rate of 50% to 90%. In April 2022, EBOV outbreak posed a serious threat to global public health in the Congo region of Africa. Using a haploid cell‐screening platform, Monteil et al. identified the guanine nucleotide exchange factor CCZ1 as a key host factor for the early replication of filoviruses. They subsequently established CCZ1 knockout cell clones using CRISPR/Cas9 and generated vascular organoids from these clones, discovering that the infection efficiency of EBOV and MARV on CCZ1‐deficient vascular organoids was indeed reduced [44]. However, bats carry a large number of viruses, including EBOV, MARV and SARS‐CoV‐2, and are natural reservoirs for various viruses, yet they do not show obvious disease symptoms themselves. Due to the difficulty of raising wild bats and the lack of representativeness in studies involving individual bats, organoid models provide a potential means to study the mechanisms of virus infection in different host‐derived organs. Elbadawy et al. successfully constructed intestinal organoids from Rousettus bats for the first time [85]. Liu et al. conducted comparative studies between bat and human intestinal organoids, finding that the baseline expression levels of IFNs and IFN‐stimulated genes in bat organoids were higher than those in humans. Under Poly(I:C) stimulation, bat organoids exhibited more rapid, robust and sustained antiviral defences compared to human organoids. When the expression of TLR3/RLR was inhibited in bat organoids, they allowed the infection and replication of SARS‐CoV‐2, suggesting that TLR3 and RLR may be conserved pathways mediating antiviral responses in bat and human intestinal organoids [86].

### 2.7 Organoid Models for Influenza Virus Research

*Influenza virus*: Influenza viruses are typically transmitted through the respiratory tract, causing influenza, which is characterised by high pathogenicity, widespread infectivity and recurrent outbreaks, making it a serious public health issue. Influenza viruses can be classified into four strains—A, B, C and D—based on differences in the antigenicity of nucleoprotein (NP) and matrix protein (MP). Among these, Type A influenza virus has the broadest host range and is the most common. Subtypes can be further divided based on the antigenicity of two viral surface glycoproteins: hemagglutinin (HA) and neuraminidase (NA), such as H1N1, H2N2, H5N1~H5N9 and H9N2. Some antiviral drugs have been applied for the treatment and prevention of influenza, such as the NA inhibitors Oseltamivir and Zanamivir, which inhibit the activity of influenza virus NA to prevent the spread of new viral particles to other cells [87]. Common types of vaccines include inactivated influenza vaccines, live attenuated influenza vaccines, and recombinant HA vaccines. Due to the high mutation rate of influenza, the development of influenza vaccines presents significant challenges, but organoid models offer new ideas for vaccine design. Researchers have found that different influenza vaccines elicit varying T/B cell responses and antibody reactions based on human tonsil organoids. The main differences in antigenic forms relate to their ability to recruit different T/B cells [33]. In addition to neutralising antibody response, studies have shown that T‐cell immune response also plays a role in controlling and eliminating the influenza virus [88]. T‐cell vaccines developed based on organoids may become the next generation of promising vaccines.

### 2.8 Organoid Models for HuNoV Research

*Norovirus*: GII.4 human norovirus (HuNoV) is a major pathogen causing acute gastroenteritis in populations worldwide, characterised by high infectivity and variability, but currently lacks effective treatment options [89]. Vijayalakshmi Ayyar et al. utilised human intestinal epithelial organoids to discover that GII.4 HuNoV entry into cells is highly dependent on cholesterol and endosomal acidification, and it invades intestinal epithelial organoids through a unique clathrin‐independent endocytosis pathway. The combined use of cholesterol inhibitors and actin polymerisation inhibitors significantly reduces the infection efficiency of HuNoV [46]. Recent studies have innovatively synthesised a recombinant rotavirus expressing the norovirus VP1 protein and P domain as a new strategy for developing vaccines against HuNoV, demonstrating its effectiveness in intestinal organoid models [47]. The ‘dual‐use’ intestinal vaccine carrier designed using rotavirus offers new hope for the prevention and treatment of HuNoV.

### 2.9 Organoid Models for HSV Research

*Herpes Simplex Virus*: Herpes Simplex Virus (HSV) includes two types: HSV‐1 and HSV‐2. HSV‐1 usually causes infections around the mouth, such as cold sores, while HSV‐2 primarily causes genital herpes. The envelope glycoproteins gC and gB of HSV can bind to heparan sulphate proteoglycans on the surface of host cells. Subsequently, the protein gD interacts with the herpesvirus entry mediator (HVEM) to initiate the fusion reaction, facilitating the entry of the virus into host cells [90]. Researchers explored the mechanism of microcephaly caused by HSV‐1 using a brain organoid model and found that HSV‐1 can efficiently replicate in brain organoids and damage the neuroepithelium. However, the organoid defects induced by HSV‐1 can be rescued by type I interferon, which blocks viral infection and replication [30]. HSV is not only highly infectious but also has a high risk of recurrence. This is because HSV can establish latent infections in sensory neurons and reactivate under immunosuppressive conditions, leading to recurrence. HSV‐1 infection causes significant changes in neuronal function and cellular transcriptomes. Although antiviral drugs such as acyclovir can inhibit viral replication in brain organoids [91], they cannot completely prevent neuronal damage caused by HSV‐1. Combining anti‐inflammatory drugs such as necrostatin‐1 or bardoxolone methyl with antiviral treatment can mitigate infection‐induced damage, suggesting that regulating acute inflammatory responses may be an effective therapeutic strategy against HSV [30]. Traditional therapies for HSV include antiviral drugs (e.g. valacyclovir [92] and acyclovir [93] in brain organoids) or vaccine development [94] (e.g. SL‐V20, HF10, VC2, mRNA‐1608). Recent studies have reported gene editing based on the delivery of HSV‐specific megacircles via adeno‐associated virus (AAV) vectors, which has emerged as a potential effective treatment method for HSV infections [95].

### 2.10 Organoid Models for HPV Research

**FIGURE 3:**
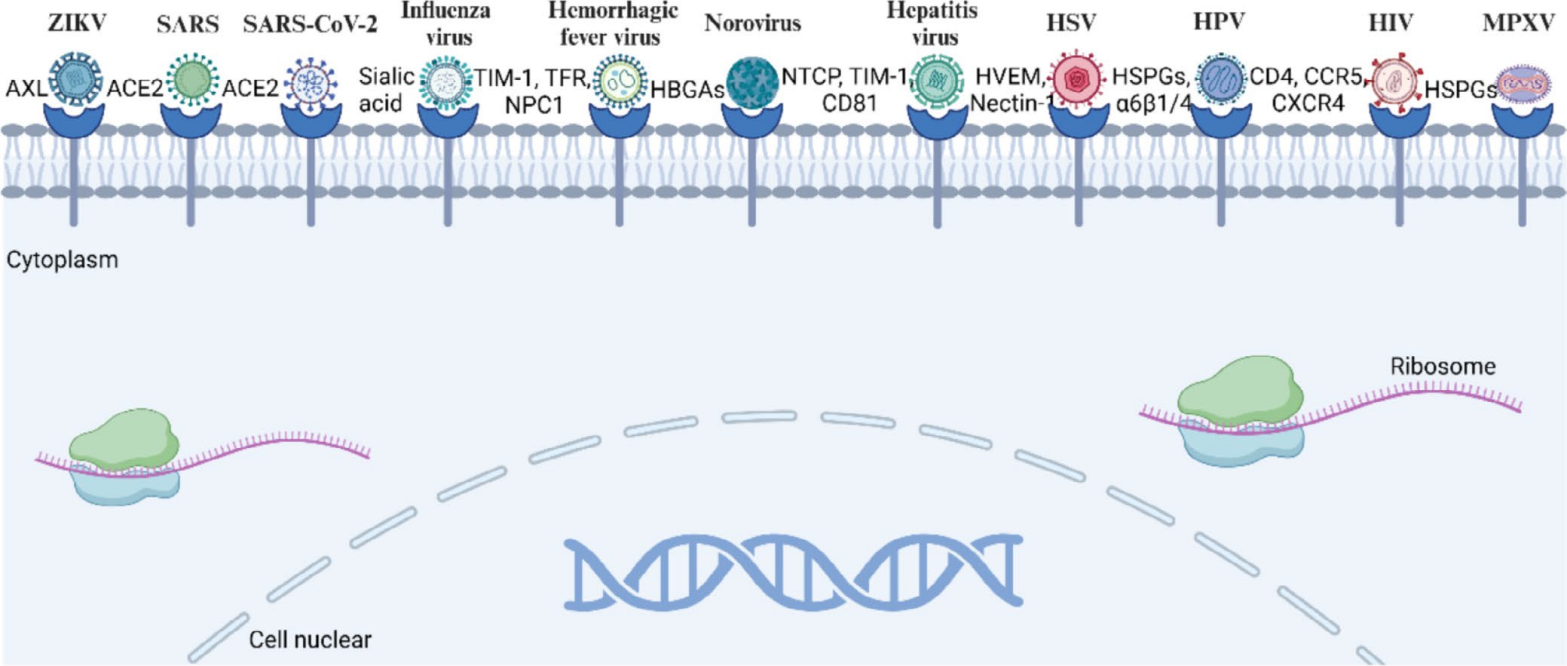
Molecular mechanisms by which different infectious disease enter cells. SARS‐CoV‐2 enters the cell through the ACE2 receptor to cause complications in the organ system. Different viruses have different ways of entering the cell. The virus that enters the cell unwraps its shell by releasing special enzymes to expose nucleic acids containing genetic material that will be used as a template to synthesise viral proteins and new nucleic acids, and the newly assembled viral particles are released outside the cell.

*Human papillomavirus*: Human papillomavirus (HPV) is a double‐stranded DNA virus from the family Papillomaviridae, classified into high‐risk and low‐risk types. High‐risk types HPV‐16 and HPV‐18 are closely associated with cervical cancer, anal cancer and oropharyngeal cancer, while low‐risk types HPV‐6 and HPV‐11 typically cause genital warts. High‐risk HPV enters basal layer cells through minor wounds or inflammatory damage, subsequently encoding the E6 protein to inhibit the normal expression of tumour suppressor genes P53 and Rb in host cells, leading to cell cycle dysregulation [96]. Cervical organoid models have shown great potential in simulating the viral dynamics of HPV and associated pathological changes. The cervical organoids constructed by researchers can stably reproduce the tissue characteristics of cervical epithelium and can simulate the integration of the HPV 16 E6 and E7 genes, developing features of precancerous lesions [97]. Exocervical organoid models have been used to study co‐infection of HPV and Chlamydia, revealing how the interactions between HPV 16 E6, E7, and Chlamydia disrupt the host's cellular repair mechanisms, thereby affecting genomic integrity and tumour progression. This provides a powerful platform for personalised treatment of cervical cancer and the development of new drugs [51]. Currently developed therapeutic HPV vaccines primarily target E6/E7 as antigenic sites, activating antigen‐specific CD8^+^ T cells to kill cells already infected with HPV, such as VGX‐3100, MEDI0457 and GX‐188E. Preventive vaccines targeting high‐risk HPV, such as Cervarix and Gardasil, have been widely used [98]. In the future, exploration of therapeutic vaccines regarding specific antibody characteristics, patient age and adjuvants will effectively reduce the transmission and infection of HPV (Table 2, Figure 3).

**TABLE 2:** Summary of pathogenic mechanism and treatment strategy of viral infectious diseases.

Virus category	Factors that regulate viral entry	Tropism	Host response	Therapeutic discovery
Human immunodeficiency virus	CCR5 in CD4^+^T lymphocytes	Lymphocytes, Microglia	Acquired immune deficiency syndrome, tumour	ART, tenofovir alafenamide fumarate, HIV protese inhibitors, rilpivirine [56]
Zika virus	Glycoprotein E [61]	Neural progenitor cell, placental trophoblast cell, testicular cell	Neural stem cell apoptosis, brain tissue injury, foetal microcephaly	Hippeastrine hydrobromide, amodiaquine dihydrochloride dihydrate [64]
Severe acute respiratory syndrome coronavirus 2	ACE2 in AT2 cell, TMPRSS2 and CTSL widely expressed, NRP1 in choroid plexus [65, 66, 67]	Choroid plexus, astrocytes, goblet cell, ciliated cell, intestinal epithelium, myocardial cell, pulmonary airway epithelium, alveolar epithelium	Excessive mucus secretion, cough, respiratory distress, diarrhoea, vomiting, headache, loss of taste, myocarditis, epilepsy	Remdesivir and the combination of ciclesonide, nelfinavir and camostat with remdesivir [72]
Monkeypox virus	Pinocytosis, membrane fusion related protein [76]	Phorocyte, colon, skin, neural progenitor, astrocyte	Fever, inflammation, skin and membrane lesions	Cidofovir, brincidofovir, tecovirimat, NIOCH‐14, ACAM2000, JYNNEOS, VGPox1/2/3 [76, 79, 80, 81]
Hepatitis virus	SR‐B1, CD81, EGFR [40]	Liver	Hepatitis, cirrhosis, liver injury	Tenofovir, brequinar, homoharringtonine [41]
Haemorrhagic fever virus	CCZ1 [44]	Intestinal tract, blood vessel	High fever, headache, diarrhoea, bleeding	—
Influenza virus	Nucleoprotein, matrix protein, hemagglutinin, neuraminidase [87]	Respiratory tract	Influenza	Oseltamivir, ranamivir [87]
Norovirus	Cholesterol, endosomal acidification, endocytic pathway [46]	Stomach, intestine	Acute gastroenteritis	—
Herpes simplex virus	GlycoproteinC/B/D, HVEM [90]	Fibroblasts, lymphocytes, neuroepithelium	Cold sores, genital herpes, microcephaly	Valacyclovir [92], acyclovir [93], SL‐V20, HF10, VC2, mRNA‐1608 [94]
Human papillomavirus	Microfissures or inflammatory lesions [96]	Cervical epithelial and basal cells	Genital warts	VGX‐3100, MEDI0457, GX‐188E, Cervarix, Gardasil [98]

## 3 The Latest Application of Organoid Model in Infectious Disease Research

**FIGURE 4:**
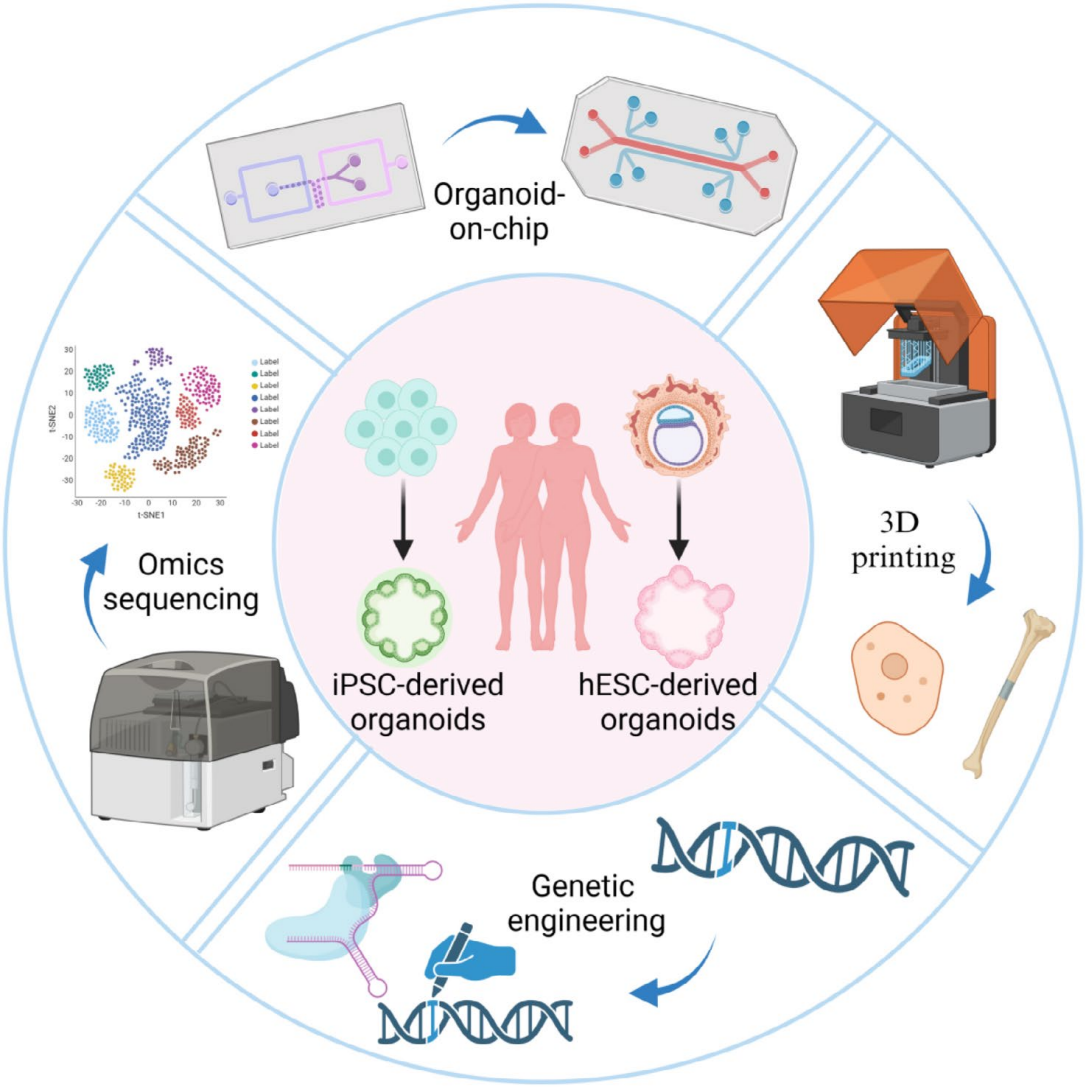
Application of new techniques to organoid models. Organoids derived from ESCs and adult stem cells are currently used in various advanced technologies, including organs‐on‐chip, 3D printing, omics sequencing and genetic engineering.

In recent years, the application of novel technologies in organoid systems has accelerated advancements in domains such as clinical disease modelling, infectious diseases and regenerative medicine. With the aid of new technologies like genetic engineering, bioprinting, microfluidics system and multi‐omics analysis, the complexity of organoids has significantly increased, facilitating the development of organoid‐based high‐throughput platforms (Figure 4).

### 3.1 Organoids and Genetic Engineering System

Using the powerful genetic tool CRISPR/Cas9, researchers are able to achieve precise knockout or knock‐in of specific genes or mutations, allowing for an in‐depth exploration of the genotype–phenotype relationship in organoids [99]. Additionally, the CRISPR/Cas9 technology possesses the capability to modify whole genomic sequences through nucleases, which gives it immense potential in engineering viral and host genomes. For example, researchers have designed multiple novel guide RNAs to target conserved motifs of HIV, effectively inhibiting HIV replication and providing insights into addressing issues of viral immune evasion or drug resistance [100]. CRISPR technology has also played an important role in the diagnosis and treatment of COVID‐19. Researchers developed a treatment method named PAC‐MAN, which specifically targets the RNA genome of SARS‐CoV‐2 to prevent further viral mutations and immune evasion, demonstrating significant advantages over vaccination [101]. Thus, the combination of organoids and gene editing technology will aid in the study of viral susceptibility as well as intercellular interactions within organoids infected with viruses.

### 3.2 Organoids and Bioprinting System

3D bioprinting technology uses bio‐ink to produce large‐scale functional 3D tissue and organoid models. Utilising low‐cost, high‐throughput 3D bioprinting platforms, complex structures such as liver organoids, lung cancer organoids and neural tissues can be efficiently generated, achieving patterned tissue formation from iPSCs into various cell types. Jian et al. successfully constructed liver organoids resembling hepatic lobules in vitro based on multicellular droplets and a layer‐by‐layer 3D printing strategy [102]. Microarray 3D bioprinting employs a column plate platform to print a mixture of hiPSC‐derived endoderm cells and Matrigel, allowing for the large‐scale generation of human liver organoids, which significantly enhances the throughput of hepatotoxic compound assessments [103]. In studies of respiratory organoids infected with SARS‐CoV‐2, 3D bioprinted airway epithelium can simulate alveolar tissue [104]. In addition, regarding biocompatibility in 3D printing, viruses can replicate within 3D models, and bio‐printed cells induce an immune response similar to that in vivo through the release of the inflammatory cytokine interleukin‐29 [105]. Using hiPSC‐based inks without extracellular matrix, researchers successfully generated patterned neural tissues with layered regions of neural stem cells, endothelial cells and neurons through multi‐material bioprinting [106]. These studies indicate that the combination of 3D bioprinting technology with organoid platforms not only overcomes the limitations of traditional methods in labour‐intensive operations and inter‐batch variability but also demonstrates significant value in disease research, drug development and personalised medicine.

### 3.3 Organoids on Microfluidics System

Organs‐on‐chip is an in vitro technology for tissue culture using microfluidic systems. In microfluidic systems, cells are arranged in a 3D manner, which not only vividly simulates the internal structures of organs but also allows for drug screening and the study of inter‐organ interactions. Hashimoto et al. reproduced the airway dysfunction observed in COVID‐19 patients using airway organoid chips [107]. Using organ‐on‐a‐chip technology, human liver organoids were co‐cultured with HLA‐matched primary CD8^+^ T cells, successfully simulating the T‐cell immune response against the HCV non‐structural protein (NS3) and enabling real‐time monitoring of T‐cell infiltration and morphological changes in the organoids [108]. Using airway organoid chips for drug screening, it was found that amodiaquine, tamoxifen and clomifene significantly reduced the number of SARS‐CoV‐2 viral particles in infected cells [109]. An ecological model of the gut‐brain axis inter‐organ interactions based on gut‐brain organ chips has already been established [110]. At the same time, cardiac micro‐tissues generated from hiPSCs were loaded with kidney organoids onto interconnected perfusion chips, allowing the maintenance of tissue viability for at least 72 h, thus providing an innovative tool for studying complex heart‐kidney interactions [111]. These studies fully demonstrate the diverse potential of microfluidic technology in organoid applications, offering important support for precision medicine and personalised treatment.

### 3.4 Organoids and Multi‐Omics Analysis

Multi‐omics sequencing is widely used to study complex organoid structures composed of various cell types. It can be utilised to investigate the interactions among different cells after viral infection and the responses of host cells to pathogens. In studies of infections with SARS‐CoV‐2 variants, multi‐omics analyses revealed that the key molecule ODC1 in the polyamine biosynthesis pathway can serve as a molecular marker for assessing viral virulence. Additionally, its differential activation was validated in human airway organoids, regulated by the renin‐angiotensin system and positively correlated with ACE2 activity [112]. In the study of viral infections and diabetes, Yang et al. conducted scRNA‐seq, spatial multi‐omics and Assay for Transposase‐Accessible Chromatin using sequencing (ATAC‐seq) analyses on human pancreatic islet cells exposed to SARS‐CoV‐2 or Coxsackievirus B4 viruses (CVB4), confirming through a vascularized macrophage‐islet organoid model that pro‐inflammatory macrophages induce β‐cell pyroptosis via the TNFSF12‐TNFRSF12A signalling pathway [48]. Multi‐omics analyses of monkeypox virus (MPXV) infections clarified the molecular mechanisms of virus‐host interactions, identified the regulatory roles of the HIPPO and TGF‐β pathways and the key functions of MAPK signalling, and discovered potential antiviral drug targets [113]. However, this study is solely based on cell lines, and many viruses lack multi‐cell type‐based omics sequencing research. The integration of organoids and multi‐omics will provide rapid and effective treatment solutions for new viral outbreaks. Therefore, the combination and application of organoid models with new technologies have led to a deeper understanding of infectious diseases, driving new frontiers in precision medicine and biomarker development.

## 4 Future Challenges and Prospects of Organoids

Organoid models not only demonstrate significant advantages in the study of infectious viruses but also possess substantial application value in research related to organ development, hereditary diseases, organ transplantation and tumours. Organoids closely mimic their source organs, effectively simulating real physiological environments. Additionally, they overcome the limitations of traditional cell and animal models while avoiding ethical concerns. However, the limitations of organoids in terms of technological optimization, standardisation of applications and industrial scaling also hinder the development of vaccines and drugs for infectious diseases. Many viral infectious diseases still lack better treatment options to this day.

In terms of technology, traditional organoid models still lack immune cells, blood vessels, neuron cells and inter‐organ communication. To address this, researchers have increased the complexity of organoids through the co‐culture of organoids with immune and endothelial cells in vitro [48]. They have integrated organoid chips and 3D printing technology to simulate physiological conditions such as blood perfusion, respiratory movements or intestinal peristalsis within the body. Continuous optimization of culturing conditions and methods for organoids is in progress to maintain long‐term stability in their growth. These efforts have initiated research on a new generation of organoids [114].

In terms of applications, the reproducibility and consistency of organoids across laboratories remain challenging due to the lack of standardised protocols. Recently, China's first national standard in the field of organ‐on‐chip technology, titled “General Technical Requirements for Skin Chips” (GB/T 44831‐2024), has been officially released. Establishing standardised protocols for the culture and testing of organoid disease models could enhance the reliability and sharing of experimental results, thereby promoting the further development of organoid technology. From an industrial perspective, there remains a considerable distance between laboratory research and clinical application. Challenges such as the use of animal‐derived components in Matrigel, high culture costs of organoids, and the demand for personalised treatment remain to be addressed. A multifunctional microfluidic chip has been developed that utilises ATP sensors to achieve comprehensive monitoring of organoid growth status, effectively supporting dynamic drug testing for lung cancer organoids and significantly enhancing screening efficiency and accuracy [115]. In 2016, the first large‐scale organoid library project, the Human Cancer Model Initiative (HCMI), was launched, and multi‐centre collaborations and clinical trials have gradually begun. Various stakeholders are actively working to advance the clinical application of organoid models.

Currently, research based on organoid models primarily focuses on vaccine and drug development. Specifically, in vaccine development, the high variability of viruses has resulted in suboptimal efficacy for many neutralising antibody vaccines. Researchers have found that, unlike antibodies targeting variable surface proteins, T‐cell responses possess a critical advantage in recognising conserved internal viral proteins, enabling cross‐reactivity against different strains [88]. The study by Nathan et al. clarified the SARS‐CoV‐2 mutation‐restricted epitopes that can be recognised by T cells, establishing a solid foundation for the development of T‐cell vaccines [116]. Wagar et al. successfully constructed a human tonsil organoid model characterised by germinal centre features, capable of simulating antigen‐specific antibody production, plasma cell differentiation and affinity maturation. Subsequently, the tonsil organoids were used to evaluate the humoral immune response to the SARS‐CoV‐2 vaccine [117]. Several T cell‐inducing influenza vaccines, such as Osivax's OVX836, Imutex's FLU‐v and Biondvax's M‐001, have shown positive results in clinical trials. It is believed that with the continuous improvement of organoid models and technologies, human ability to defend against new emerging infectious diseases will be greatly enhanced.

In terms of drug development, in August 2022, the FDA approved a new drug (NCT04658472) that is entirely based on ‘organoid chip’ research to enter clinical trials. This marks the first time globally that a drug has been approved for clinical trials without utilising animal testing, representing a milestone event in the development of organoids and new drug research and development [118]. Therefore, antiviral drug research based on organ‐on‐chip technology is thriving. The Savas Tay team developed an automated microfluidic platform for dynamic and combinatorial drug screening of human pancreatic tumour organoids. They found significant differences in the drug treatment responses of organoids derived from different patients. This high‐throughput, high‐dynamic and real‐time monitoring automated organoid microfluidic platform provides strong support for drug development [119]. In a multi‐organoid‐on‐a‐chip system based on the co‐culture of liver and cardiac organoids derived from hiPSCs, the liver organoids demonstrate robust capabilities for albumin and urea synthesis, while the cardiac organoids exhibit normal contractile functions. This system is capable of simulating the hepatic metabolic processes of drugs in vivo and assessing their potential cardiotoxicity. The interactions between different organoids are crucial for evaluating the safety of drugs following hepatic metabolism in vitro [120]. With continuous technological optimization, organoid models are poised to advance precision medicine, regenerative therapies and drug development, offering vast potential for future infectious disease applications.

## Author Contributions

Sijing Zhu, Dan Chen and Yuling Han wrote the manuscript and drew the tables and pictures. Xinzhi Yang and Liuliu Yang were in charge of literature search and manuscript correction.

## Conflicts of Interest

The authors declare no conflicts of interest.

## Data Availability

Data sharing is not applicable to this article as no datasets were generated or analyzed during the current study.
